# Partial Purification of Integral Membrane Antigenic Proteins from *Trypanosoma evansi* That Display Immunological Cross-Reactivity with *Trypanosoma vivax*

**DOI:** 10.1155/2014/965815

**Published:** 2014-03-17

**Authors:** Norma P. Velásquez, Rocío E. Camargo, Graciela L. Uzcanga, José Bubis

**Affiliations:** ^1^Departamento de Química, Universidad Simón Bolívar, Apartado 89.000, Valle de Sartenejas, Caracas 1081-A, Venezuela; ^2^Departamento de Biología Celular, Universidad Simón Bolívar, Apartado 89.000, Valle de Sartenejas, Caracas 1081-A, Venezuela; ^3^Dirección de Salud, Fundación Instituto de Estudios Avanzados (IDEA), Valle de Sartenejas, Caracas 1015-A, Venezuela; ^4^Laboratorio de Química de Proteínas, Departamento de Biología Celular, División de Ciencias Biológicas, Universidad Simón Bolívar, Apartado 89.000, Valle de Sartenejas, Baruta, Caracas 1081-A, Venezuela

## Abstract

*Trypanosoma evansi* and *Trypanosoma vivax*, which are the major causative agents of animal trypanosomosis in Venezuela, have shown a very high immunological cross-reactivity. Since the production of *T. vivax* antigens is a limiting factor as this parasite is difficult to propagate in experimental animal models, our goal has been to identify and isolate antigens from *T. evansi* that cross-react with *T. vivax*. Here, we used the Venezuelan *T. evansi* TEVA1 isolate to prepare the total parasite lysate and its corresponding cytosolic and membranous fractions. In order to extract the *T. evansi* integral membrane proteins, the particulate portion was further extracted first with Triton X-100, and then with sodium dodecyl sulfate. After discarding the cytosolic and Triton X-100 solubilized proteins, we employed sedimentation by centrifugation on linear sucrose gradients to partially purify the sodium dodecyl sulfate-solubilized proteins from the Triton X-100 resistant particulate fraction of *T. evansi*. We obtained enriched pools containing polypeptide bands with apparent molecular masses of 27 kDa, 31 kDa, and 53 kDa, which were recognized by anti-*T. vivax* antibodies from experimentally and naturally infected bovines.

## 1. Introduction

Mammal-infecting* Trypanosoma* species are grouped into the sections Stercoraria and Salivaria. Trypanosomes classified as Stercoraria develop in the posterior part of the vector digestive tract and are transmitted through feces. In contrast, the biological transmission of trypanosomes classified as Salivaria occurs by insects that harbor the parasites in their salivary glands, although mechanical transmission also occurs. Salivarian trypanosomes include the subgenera* Tejeraia* (*Trypanosoma rangeli*),* Duttonella* (*Trypanosoma vivax*),* Nannomonas* (*Trypanosoma congolense*),* Trypanozoon* (*Trypanosoma brucei, Trypansoma evansi*, and* Trypanosoma equiperdum*), and* Pycnomonas* (*Trypanosoma suis*).

Cross-reactions among evolutionarily close parasites are generally explored either to prevent false positive interpretation of the tests [1, 2] or to take advantage of them through an interspecific detection assay [3]. Antigenic similarities among salivarian trypanosomes have been known for a long time; indeed cross-reactions between* T. congolense*,* T. vivax*,* T. evansi,* and* T. brucei *spp. have been repeatedly reported [4–6]. Even monoclonal antibodies developed for the diagnosis of specific salivarian trypanosome species have been shown to cross-react all together [7, 8]. Recently, similarities between salivarian and stercorarian trypanosomes have also been recorded [9], showing an important cross-reactivity between* T. evansi* and* T. cruzi*.

*T*.* vivax* and* T*.* evansi* are salivarian parasite species that cause animal trypanosomosis predominantly in bovines and equines, respectively. They are the major causative agents of animal trypanosomosis in Venezuela. Given that it is more convenient to produce* T. evansi* which grows easily in rodents, than* T. vivax* which does not, the use of* T. evansi* crude antigen or purified cross-reacting antigenic proteins for the detection of* T. vivax* infections in cattle has been under investigation for the last two decades [3, 10–12]. Moreover, the use of* T. evansi* antigens in the diagnosis of* T. vivax* infections is an interesting alternative for laboratories which do not have the facilities to produce* T. vivax* antigens. Since the parasite cell surface is an intuitive place to explore for antigenic potential proteins, we partially purified sodium dodecyl sulfate- (SDS-) solubilized membrane-associated integral proteins from the Venezuelan* T. evansi* TEVA1 isolate,* aka* TeAp-N/D1 [13], by using sedimentation by centrifugation on linear sucrose gradients. We obtained fractions containing* T. evansi* polypeptides with apparent molecular weights of 27,000, 31,000, and 53,000, which were recognized by anti-*T. vivax* antibodies from infected cows.

## 2. Materials and Methods

### 2.1. Materials

Reagents were purchased from the following sources: anti-mouse IgG horseradish peroxidase conjugate, middle range molecular weight protein markers, Promega; anti-rabbit IgG horseradish peroxidase conjugate, anti-bovine IgG horseradish peroxidase conjugate, anti-bovine IgG alkaline phosphatase conjugate, anti-equine IgG alkaline phosphatase conjugate, diaminobenzidine (DAB), fibrous DEAE-cellulose, benzamidine, iodoacetamide, L-trans-epoxysuccinyl-leucylamido(4-guanidino)butane (E-64), leupeptin, phenyl methyl sulfonyl fluoride (PMSF), Sigma; prestained high molecular weight protein markers, Gibco BRL; 5-bromo-4-chloro-3 indolyl phosphate (BCIP), nitro blue tetrazolium (NBT), protein assay dye reagent concentrate, bovine serum albumin (BSA), broad range molecular weight standards, and nitrocellulose membranes (0,45 *μ*m pore size), BioRad. All other chemicals were of the highest quality grade available.

### 2.2. Source of Antigens

We used a Venezuelan field isolate of* T. evansi* named TEVA1 [13]. Cryopreserved* T. evansi*-infected blood was inoculated intraperitoneally into adult albino rats (Sprague-Dawley). When the number of parasites reached ≥10^6^ trypanosomes/mL, the blood was extracted from the rats by cardiac puncture using 0.5 M EDTA as anticoagulant. Parasites were purified by anion exchange chromatography using a fibrous DEAE-cellulose column [14] and were kept frozen at −80°C until further use.

### 2.3. Isolation of the* T. evansi* Particulate Fraction

Purified* T. evansi* parasites (~5 × 10^9^) were extracted on ice, by sonication (4 cycles of 7 watts, 30 sec each, with a 2 min resting period in between) using 16 mL of lysis buffer (5 mM Tris-HCl buffer (pH 8.0) containing 1 mM benzamidine, 1 mM PMSF, 10 mM EDTA, 10 mM EGTA, 1 mM iodoacetamide, 20 *μ*M E-64, and 20 *μ*M leupeptin). The parasite homogenate (H) was centrifuged at 100,000 ×g for 30 min, at 4°C, to separate the pellet containing the particulate fraction (P) from the supernatant holding the clarified soluble fraction (S). This procedure was repeated three times to completely wash all soluble antigens from the remaining parasite membranous fraction. In an attempt to solubilize this fraction, P was subjected to three successive extractions with lysis buffer containing 2% Triton X-100. A centrifugation step at 100,000 ×g for 30 min, at 4°C, was employed to separate the supernatant (S_TX-100_) from the neutral detergent-washed pellet (P_TX-100_).

### 2.4. Solubilization with SDS of the Triton X-100 Washed Parasite Particulate Fraction

The Triton X-100 washed pellet was resuspended in lysis buffer containing 4% SDS and homogenized by passage through a number 23 needle. A centrifugation step at 100,000 ×g for 30 min, at room temperature, was employed to separate the supernatant (S_SDS_) from the anionic detergent washed pellet (P_SDS_). The concentration of SDS was reduced to 2% in the resulting supernatant sample, before assaying its polypeptide and antigenic composition by SDS-polyacrylamide gel electrophoresis (SDS-PAGE) and Western blot, respectively.

### 2.5. Partial Purification of Antigens from the* T. evansi* SDS-Solubilized Particulate Fraction Using Zonal Sedimentation on Sucrose Gradients

Linear 10–30% and 5–20% sucrose analytical gradients (4 mL) were prepared in 100 mM Tris-HCl (pH 8.0), 0,2 mM EDTA, 0,4 mM dithiothreitol, 10 mM MgCl_2_, 0.3 M NH_4_Cl, and 2% SDS. After loading the parasite SDS-solubilized particulate fraction sample onto the sucrose gradients, the tubes were centrifuged at 200,000 ×g, at room temperature, for 18 h. Fractions were collected through the bottom of the tube, and an aliquot of each fraction was analyzed by SDS-PAGE and Western blot using sera from* T. evansi*-infected horses and* T. vivax*-infected cows.

### 2.6. Animal Sera

Two healthy horses were experimentally infected with cryopreserved blood samples containing ~10^6^ parasites of different* T. evansi* isolates. The first horse was inoculated with the TEVA1* T. evansi* isolate (H-TEVA1), and the second horse was inoculated with the TeAp-ElFrio01 isolate [13] (H-TeApEF). Three healthy bovines were infected with a cryopreserved blood sample containing ~10^6^ parasites of the LIEM-176* T. vivax *isolate [15] (B-103, B-173, and B-303). Blood samples from the experimentally infected animals were taken every day, for a two-month period, and sera were stored frozen at −20°C and used as positive controls.  Table 1 summarizes the samples of sera obtained from experimentally infected animals.

In addition, blood samples were collected from the jugular vein of parasitologically negative and trypanosome-infected field animals. Horses and cows were examined for the presence of trypanosomes in the circulation by the microhematocrit technique [16] and were diagnosed as positive or negative for trypanosomosis by indirect ELISA, using the clarified antigenic fraction from* T. evansi* as the source of antigen [10, 17]. Following blood clotting at room temperature and centrifugation, each serum was stored frozen at −20°C and employed for immunodetection.  Table 2 summarizes the samples of sera obtained from field animals.

### 2.7. Other Procedures

Protein concentration was measured according to Bradford [18], using bovine serum albumin as protein standard. The p64 antigen, which is the soluble form of a* T. evansi* variant surface glycoprotein (VSG) that displays cross-reactivity between* T. evansi* and* T. vivax, *was purified as described by Uzcanga et al. [11]. The purified p64 was used to produce polyclonal antibodies in mice ascitic fluid [12]. Polyclonal anti-VSG antibodies were also generated in rabbit serum, following the procedure described by Harlow and Lane [19]. SDS-PAGE was carried out on 1.5 mm thick slab gels containing 12 or 15% polyacrylamide [20]. Coomassie blue R-250 was used for protein staining. For Western blot analyses; the proteins were electrotransferred from the gels to nitrocellulose filters [21]. For immunodetection, the filters were incubated with bovine sera (dilution 1 : 100) or specific anti-VSG polyclonal antibodies generated in mice (dilution 1 : 5,000) or rabbits (dilution 1 : 150). The sheets were then incubated with the appropriate dilution of alkaline phosphatase-conjugated or horseradish peroxidase-conjugated secondary antibodies against bovine, mouse, or rabbit IgG, depending on the case, following the instructions of the supplier. Finally, the polypeptide bands were visualized by the addition of NBT and BCIP when alkaline phosphatase-conjugated antibodies were used, or DAB and hydrogen peroxide when horseradish peroxidase-conjugated antibodies were employed, according to the provider. A lane containing a mixture of molecular weight protein markers was included in the blot to determine the apparent size of the polypeptide bands.

## 3. Results

**Figure 1:**
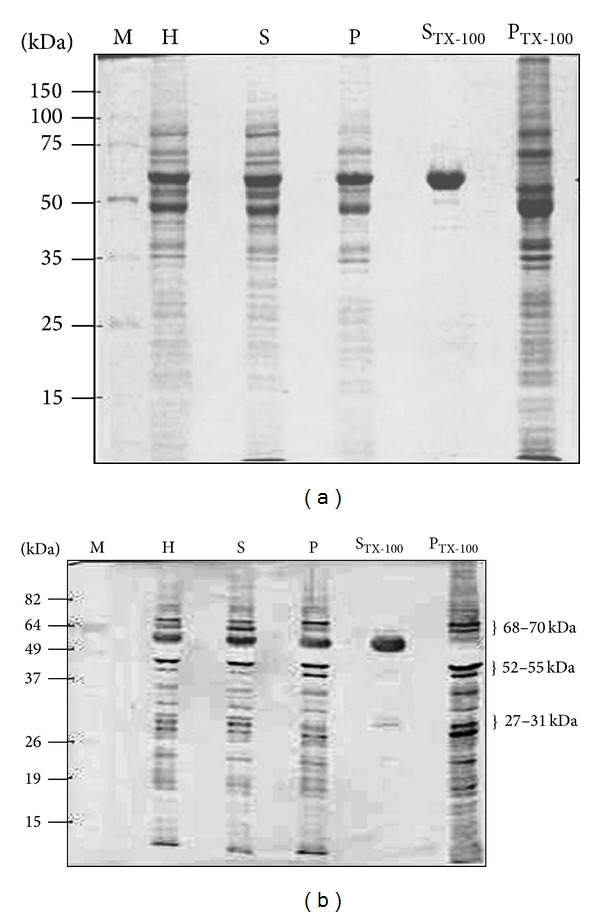
Analysis of* T. evansi* antigenic polypeptide bands that display cross-reactivity with* T. vivax*. Purified* T. evansi* parasites were homogenized to generate the whole-cell extract (H), which was centrifuged to separate the soluble fraction (S) from the particulate fraction (P). Then, P was extracted with 2% Triton X-100, and the Triton X-100 solubilized fraction (S_TX-100_) was separated from the remaining pellet (P_TX-100_) by centrifugation. (a) Coomassie blue staining. (b) Immunoblot developed with serum B-303 (Table 1). M = protein molecular weight markers.

Figure 1(a) illustrates the Coomassie blue stained polypeptide profiles, which were acquired following SDS-PAGE separation of various* T. evansi* fractions (H, S, P, S_TX-100_ and P_TX-100_). As seen in the figure, a variety of polypeptide bands possessing a broad range of sizes were obtained in the different fractions. Then, serum from a bovine experimentally infected with* T. vivax* was used to identify the TEVA1* T. evansi* antigenic polypeptides that exhibited cross-reactivity with* T. vivax *by Western blotting. As previously reported [10], a series of cross-reacting antigens with apparent molecular masses ranging from approximately 10,000 to 110,000 daltons were evident in the* T. evansi* homogenate (Figure 1(b)). Both* T. evansi* fractions, S and P, also contained antigens that display cross-reactivity with* T. vivax *(Figure 1(b)). Moreover, a 64 kDa polypeptide band (p64) was the major polypeptide species of* T. evansi*, which was present in the H, S, and P fractions, and was recognized by bovine anti-*T. vivax* antibodies. Interestingly, the S_TX-100_ sample was highly enriched with the 64 kDa polypeptide band that exhibited cross-reactivity with* T. vivax*, but no p64 was observed in the P_TX-100_ sample (Figure 1(b)). Additionally, a series of cross-reacting antigenic polypeptide bands were resistant to the neutral detergent treatment and remained in the P_TX-100_ sample. The resulting pattern showed that the most intensive antigenic bands corresponded to polypeptide bands migrating at about 68–70 kDa, 52–55 kDa, and 27–31 kDa, respectively (Figure 1(b)).

**Figure 2:**
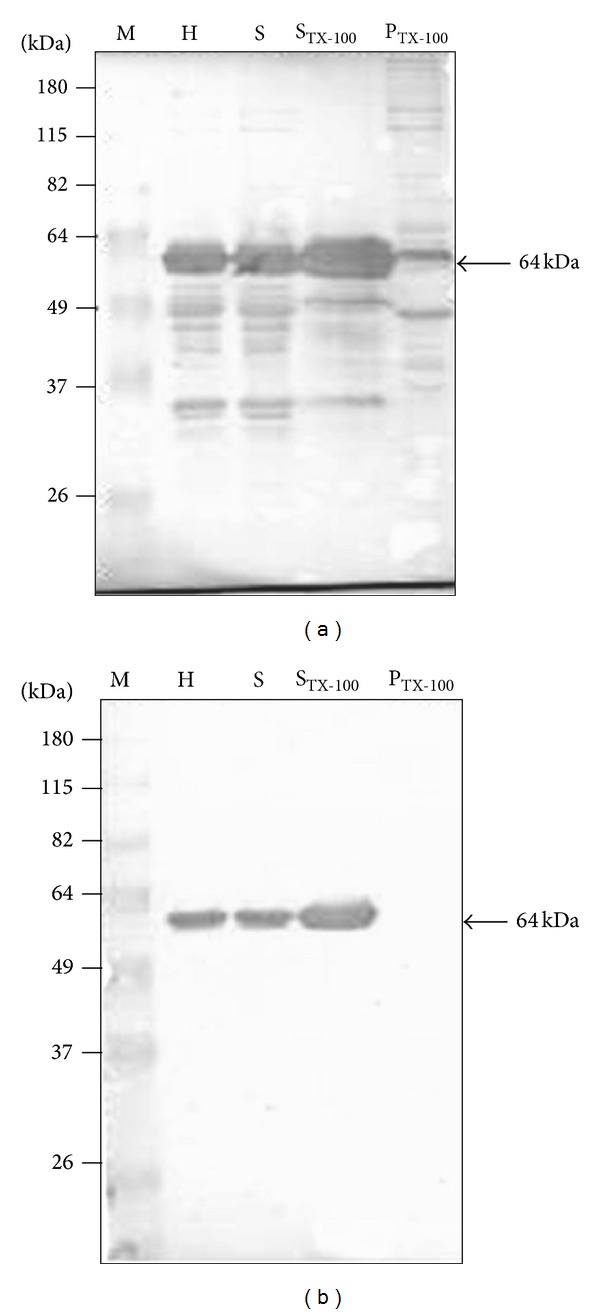
The 64 kDa band that is enriched in the S_TX-100_ fraction corresponds to the previously reported p64 antigen [10, 11], that is partially responsible for the cross-reaction between* T. evansi* and* T. vivax*. The p64 antigen was observed in the parasite homogenate (H), soluble (S), and Triton X-100 solubilized (S_TX-100_) fractions. No p64 was obtained in the resulting pellet after treatment with Triton X-100 (P_TX-100_). (a) Western blot using polyclonal anti-p64 antibodies prepared in rabbit sera. (b) Western blot using polyclonal anti-p64 antibodies prepared in mice ascitic fluid. M = protein markers.

**Figure 3:**
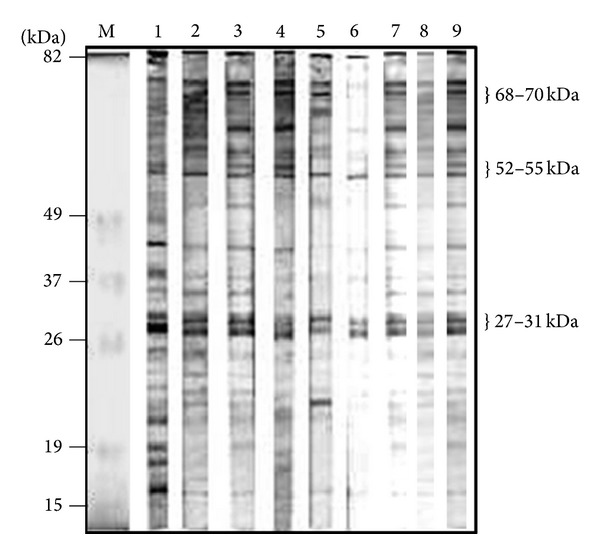
Identification of* T. vivax*-cross-reacting antigens from the P_TX-100_ fraction of* T. evansi*. An aliquot of the P_TX-100_ fraction (350 *μ*g of total protein) was separated by electrophoresis on a preparative 15% polyacrylamide slab gel. Following SDS-PAGE, the proteins were electrotransferred to nitrocellulose, and the blot was cut into 3 mm strips. Strips containing the P_TX-100_ fraction were developed using sera B-303 (lane 1), B-LC31 (lane 2), B-LE14 (lane 3), B-P13 (lane 4), B-LC43 (lane 5), B-LL19 (lane 6), H-TEVA1 (lane 7), H-TeApEF (lane 8), and H-N/D (lane 9). Sera are described in Tables 1 and 2.

Previously, Uzcanga et al. [11] demonstrated that the predominant 64 kDa cross-reacting antigen between* T. evansi* and* T. vivax*, which was purified from the TEVA1* T. evansi* Venezuelan isolate, represented the soluble form of a VSG. The 64 kDa band obtained in the H, S, P, and S_TX-100_ parasite fractions was identified here as the soluble form of the same VSG (p64), by using specific anti-VSG polyclonal antibodies that were raised in rabbits and mice (Figure 2). As expected from the results shown in Figure 1(b), no VSG remained in the P_TX-100_ fraction following treatment with Triton X-100 (Figure 2). Interestingly, several of the polypeptide bands that remained in the P_TX-100_ fraction, including the polypeptides of approximately 68–70 kDa, 52–55 kDa, and 27–31 kDa, were immunorecognized by sera from bovines experimentally and naturally infected with* T. vivax* (Figure 3). These results demonstrated that these cross-reacting antigenic bands are parasite integral proteins that are probably localized in Triton X-100 resistant membrane microdomains. As also seen in the figure (lanes 7–9), sera from horses experimentally and naturally infected with* T. evansi* recognized the same antigenic polypeptide bands than anti-*T. vivax* antibodies.

**Figure 4:**
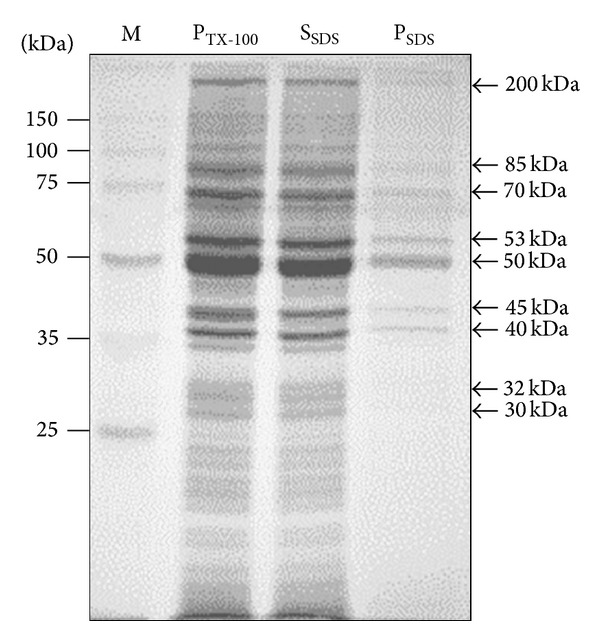
Solubilization of the proteins contained in the P_TX-100_ fraction using SDS. The* T. evansi *P_TX-100_ fraction was homogenized using 4% SDS. Following centrifugation, the supernatant (S_SDS_) was separated from the SDS-washed pellet (P_SDS_). The concentration of SDS was reduced to 2% and the polypeptide composition of the S_SDS_ and P_SDS_ fractions was evaluated by SDS-PAGE. Shown is the Coomassie blue staining of the gel. M = protein molecular weight standards.

Since SDS is a strong anionic detergent, which is capable of disintegrating membrane structures and denaturing most integral membrane proteins, the P_TX-100_ fraction was reextracted with lysis buffer containing 4% SDS, in order to solubilize its antigenic proteins.  Figure 4 shows a separation by SDS-PAGE of the polypeptide bands that were released with the SDS treatment (S_SDS_), and those that remained in the final pellet (S_SDS_), following centrifugation. In the presence of 4% SDS, most proteins were solubilized (see the S_SDS_ fraction), and a clear decrease in the amount of polypeptide bands was evident in the P_SDS_ sample (Figure 4). Polypeptides with apparent molecular masses ranging from <25,000 to >150,000 Da were observed in the* T. evansi *S_SDS_ fraction (Figure 4). The sizes of the major polypeptide bands contained in the parasite S_SDS_ fraction are indicated in the figure.

**Figure 5:**
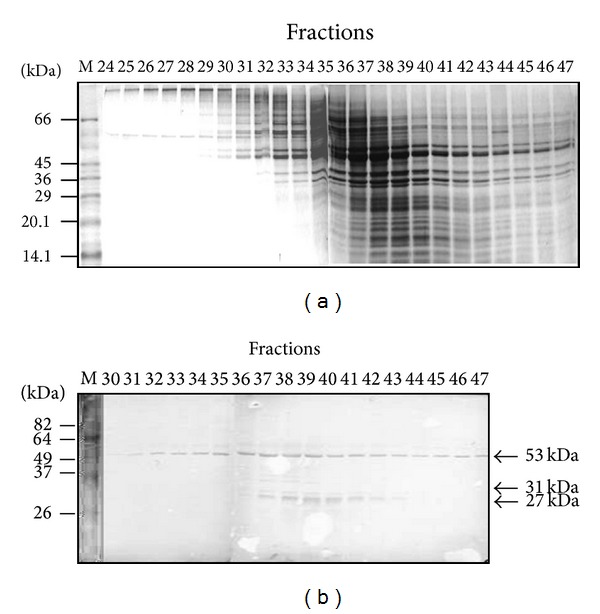
Separation of the S_SDS_ fraction using sedimentation on a linear 10–30% sucrose gradient. A 200 *μ*L aliquot of the* T. evansi *S_SDS_ sample was loaded on a 4 mL linear 10–30% sucrose gradient. Fractions (80 *μ*L) were collected through the bottom of the tube following ultracentrifugation. (a) SDS-PAGE analysis of the resulting fractions; shown is the Coomassie blue staining. (b) Western blot developed with serum B-173 (Table 1). M = protein molecular weight markers.

In order to separate the* T. vivax*-cross-reacting antigens, the* T. evansi* SDS-solubilized particulate fraction was subjected to ultracentrifugation by zonal sedimentation on sucrose gradients. A 200 *μ*L aliquot of the S_SDS_ sample was loaded on a 4 mL linear 10–30% sucrose gradient. Following centrifugation, 47 fractions of 80 *μ*L each were collected through the bottom of the tube. An aliquot of each fraction was analyzed by SDS-PAGE and Western blot using serum from a naturally* T. vivax*-infected bovine. The bulk of the proteins were separated between fractions 24 and 47 (Figure 5), and almost no proteins were seen in the first 23 fractions (data not shown). As illustrated in the figure by SDS-PAGE, the majority of the sedimented polypeptide bands were of low and middle apparent molecular weights. Western blot analysis showed a major cross-reacting polypeptide band of 53 kDa that was present in all the protein containing fractions (Figure 5). The cross-reacting antigen of 53 kDa showed a peak between fractions 37 and 39. Although fractions 30 to 36 were contaminated with other nonantigenic polypeptide bands, the only cross-reacting antigen contained in these fractions was the 53 kDa polypeptide band. Particularly, fractions 35 and 36 were enriched in the 53 kDa cross-reacting antigenic band (Figure 5). In addition, cross-reacting antigenic bands of 31 and 27 kDa were also obtained between fractions 37 and 43, showing a peak in fractions 39 and 40 (Figure 5).

**Figure 6:**
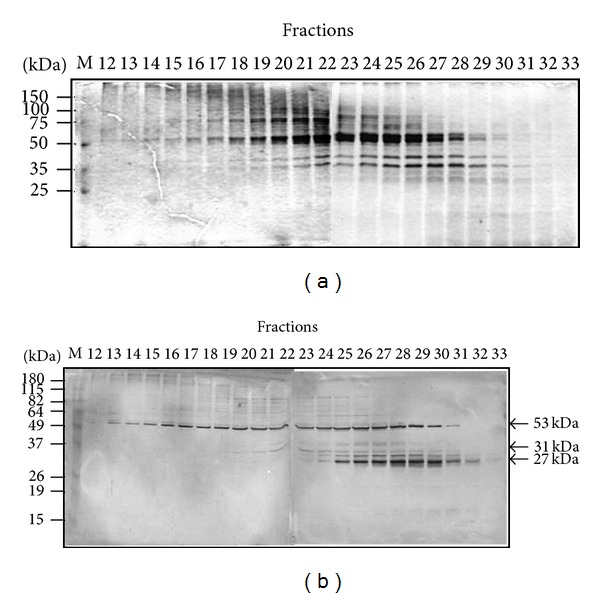
Separation of the S_SDS_ fraction using sedimentation on a linear 5–20% sucrose gradient. A 200 *μ*L aliquot of the* T. evansi *S_SDS_ sample was loaded on a 4 mL linear 5–20% sucrose gradient. Fractions (110 *μ*L) were collected through the bottom of the tube following ultracentrifugation. (a) SDS-PAGE analysis of the resulting fractions; shown is the Coomassie blue staining. (b) Western blot developed with serum B-173 (Table 1). M = protein molecular weight markers.

Given that no proteins were obtained in the first 23 fractions of the 10–30% sucrose gradient, a 4 mL linear 5–20% sucrose gradient was also employed in order to improve the antigenic separation. Following centrifugation, 41 fractions of 110 *μ*L each were collected through the bottom of the tube and were analyzed as above. Western blot analysis showed that the major 53 kDa cross-reacting antigen eluted between fractions 13 and 31 and showed a peak around fraction 28 (Figure 6). The cross-reacting antigenic bands of 27 and 31 kDa were observed between fractions 25 and 32, showing a peak around fractions 29-30 (Figure 6). In conclusion, fractions enriched in three predominant antigens with apparent molecular masses of 27, 31, and 53 kDa, which are partially responsible of the cross-reactivity between* T. vivax* and* T. evansi*, were obtained from the Triton X-100 resistant particulate fraction of* T. evansi*. Moreover, an improvement on the separation of these cross-reacting antigens was obtained when the sedimentation was carried out on a linear 5–20% sucrose analytical gradient (Figure 6).

**Figure 7:**
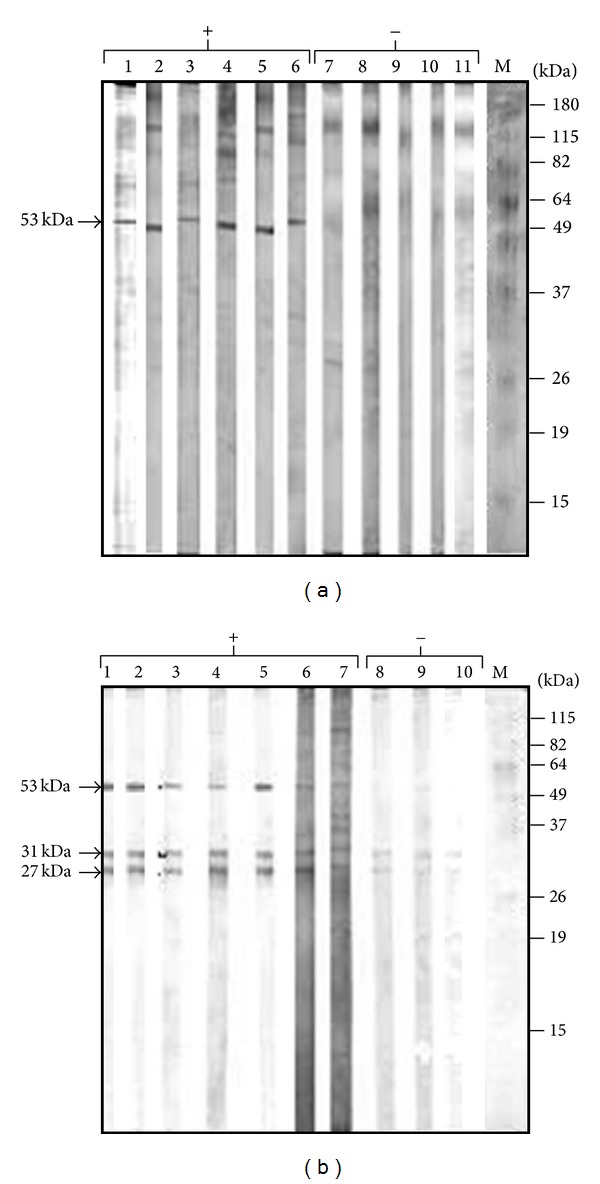
Reactivity of the* T. evansi* polypeptide bands contained in Pools I and II against sera from bovines experimentally and naturally infected with* T. vivax*. Aliquots (400 *μ*L) of Pools I and II (fractions 13–19 and 25–33 from Figure 6, resp.) were separated by electrophoresis on a preparative 12% polyacrylamide slab gel. Following SDS-PAGE, the proteins were electrotransferred to nitrocellulose filters, and the blots were cut into 3 mm strips. Strips containing Pool I (a) or Pool II (b) were developed using positive sera (+) from horses experimentally infected with* T. evansi *or from bovines experimentally or naturally infected with* T. vivax*. Parasitologically and serologically negative sera (−) were also employed as controls. In (a), lanes 1–11, sera H-TEVA1, H-TeApEF, B-303, B-103, B-173, B-LC12, B-LC29, B-LC69, B-F5315, B-F5683, and H-LR, respectively. In (b), lanes 1–10, sera H-TEVA1, H-TeApEF, B-303, B-103, B-173, B-LC12, B-LC54, B-F5315, B-F5683, and H-LR, respectively. Sera are described in Tables 1 and 2.

We pooled fractions 13–19 from the 5–20% sucrose gradient since they only contained the 53-kDa cross-reacting antigenic band (Pool I). Additionally, we also pooled fractions 25 to 33 (Pool II), which contained a mixture of all major cross-reacting bands (27, 31, and 53 kDa polypeptides). The cross-reactivity of the partially purified antigens from each pool was analyzed by Western blot employing different sera from bovines experimentally or naturally infected with* T. vivax*. Aliquots of 400 *μ*L of Pool I and Pool II were loaded on 12% polyacrylamide preparative gels. Once, the separation was finished, the gels were electrotransferred to nitrocellulose membranes, which were cut into strips and analyzed using various sera from* T. vivax*-infected bovines. As shown in Figures 7(a) and 7(b) (Pools I and II, resp.), the partially purified antigens of 27, 31, and 53 kDa were recognized by sera from horses infected with the TEVA1 and the TeAp-ElFrio01* T. evansi* isolates and from cows experimentally and naturally infected with* T. vivax*. Hence, the polypeptides of 27, 31, and 53 kDa correspond to* T. evansi* antigens that exhibit cross-reactivity with* T. vivax*.

## 4. Discussion

Absolute control of bovine trypanosomosis cannot be achieved with the methods that are currently available, which are inadequate to prevent the enormous economic impact caused by this disease. Early and accurate diagnosis of the trypanosomosis caused by* T. vivax* is of paramount importance for the strategic use of the available antiparasitic drugs. However, the diagnosis of this disease may be difficult as there are no pathognomonic clinical signs of infection, and standard trypanosome detection methods are not sensitive enough. Bovine trypanosomosis is detected using parasitological (microhematocrit centrifugation procedure) [16], immunological (indirect immunofluorescence, indirect ELISA, Western blot, etc.), and molecular (polymerase chain reaction or PCR) diagnostic techniques. In particular, the immunological tests are valuable methods for* T. vivax* diagnosis because of their high sensitivity in detecting antibodies to trypanosome antigens. Despite advances in the diagnosis of animal trypanosomosis, many acute infections go unnoticed and a chronic form of disease, frequently with no parasitemia, is more prevalent [22].

As in other infectious diseases, the early diagnosis of animal trypanosomosis is essential before local outbreaks become an epidemic of substantial proportions. This is particularly fundamental in bovine farms, where the disease can be transmitted mechanically by the vector flies (Tabanids) from one host to another, with the possibility of reaching the whole herd in a very short time. Woo [23] has shown that the magnitude of enzootic trypanosomosis caused by non-tsetse borne* T. evansi* is about three times greater than due to tsetse borne trypanosomes. Thus, cross-reactions among evolutionarily close parasites can be capitalized by using an interspecific detection assay. In particular, cross-reaction has been extensively reportedamong the immunogenic components of* T*.* vivax* and* T*.* evansi*. A predominant 64 kDa glycosylated cross-reacting antigen, p64, was previously purified from the TEVA1* T. evansi* isolate and identified as the soluble form of a VSG [10, 11]. We have also purified two additional proteins with native molecular masses of approximately 51 and 68 kDa from the cytosolic fraction of the same* T. evansi* isolate, which were proven to be recognized by anti-*T. vivax* bovine antibodies and were not related to the purified p64 [12]. In the present work, we have continued with the isolation and characterization of the antigenic proteins from* T. evansi*, which are partially responsible for the immunological cross-reaction with* T. vivax*.

Similar to the bloodstream forms of* T. brucei*,* T. evansi* contains a glycosylphosphatidylinositol-specific phospholipase C (GPI-PLC). This enzyme cleaves the GPI-anchor of the VSG, forming free diacylglycerol in the membrane and, probably, a 1,2-cyclic phosphate on the inositol ring, which remains attached to the released VSG [24, 25]. This cleavage converts the membrane-bound form of the VSG (mVSG) to the soluble released form of the VSG (sVSG) [26]. As demonstrated in Figure 2 for p64, the GPI-PLC from the TEVA1* T. evansi* isolate is active and functional. Then, other parasite GPI-anchored cell surface proteins must also be cleaved by the parasite GPI-PLC enzyme throughout the various extraction steps performed here, yielding their corresponding soluble released forms. Our results revealed that three integral membrane-associated polypeptide bands from* T. evansi*, which do not contain GPI anchors and with apparent molecular masses of 27, 31, and 53 kDa, contained common epitopes with* T. vivax *proteins. Consequently, these common or highly conserved antigenic polypeptides from* T. evansi* are good candidates to be considered as tools for immunodiagnosis of the trypanosomosis caused by* T. vivax*.

The subgenus* Trypanozoon* is the most homogeneous group of salivarian trypanosomes, which contains three recognized species that are morphologically indistinguishable,* T. brucei*,* T. evansi*, and* T. equiperdum*. The typical classification of* T. brucei*,* T. equiperdum*, and* T. evansi* as separate species is based on differences in the mode of transmission, host range and pathogenicity, and longstanding understanding that* T. brucei* contains interlocked maxicircle and minicircle kinetoplast DNA molecules (kDNA),* T. equiperdum* retains at least a part of maxicircle kDNA (dyskinetoplastidy), and* T. evansi* completely loses it (akinetoplastidy) [27–29]. There is a widely accepted paradigm that holds that* T. evansi* evolved, via* T. equiperdum*, when camels infected with* T. brucei* moved to tsetse-free areas [30], and a recent study has even suggested that* T. equiperdum* and* T. evansi* can be classified as subspecies of* T. brucei *[31]. Consequently,* T. brucei*,* T. evansi*, and* T. equiperdum* are phylogenetically very close. Although the genome of* T. evansi* has not been solved yet, the genome of a taxonomically relative parasite,* T. brucei brucei *(strain 927), which causes African sleeping sickness in humans, is now complete [32], providing both a milestone for trypanosome biology and an opportunity to consider a multitude of questions at the genome level. More recently, Jackson et al. [33] have produced high-quality draft genome sequences for two related African trypanosomes,* T. congolense *(subgenera* Nannomonas*) and* T. vivax *(subgenera* Duttonella*), specifically* T. congolense *IL3000 and* T. vivax* Y486. All these genome sequences are accessible through GeneDB (http://www.genedb.org/) or TriTrypDB (http://tritrypdb.org/). In an attempt to identify plausible candidates for the three* T. vivax*-cross-reacting antigens with apparent molecular masses of 27, 31, and 53 kDa, which were found in the Triton X-100 resistant membrane fraction of* T. evansi*, we analyzed the* T. brucei brucei* genome searching for gene products that (i) were putative integral proteins, (ii) were identified as immunodiagnostic antigens, and (iii) possessed similar sizes.

Sullivan et al. [34] took a nonbiased proteomic approach to identify potential diagnostic antigens for human African trypanosomosis, by asking which* T. brucei* proteins bind to the antibodies in sera of* T. brucei gambiense*-infected patients and not to the antibodies of uninfected individuals. This approach provided a list of twenty-four trypanosome proteins that selectively bound to the antibodies of infected patients and that might, therefore, be considered as immunodiagnostic antigens. In the same year, Jackson et al. generated a cell surface phylome for African trypanosomes, by comparing genes predicted to encode cell surface proteins of* T. brucei* with those from* T. congolense *and* T. vivax *(http://www.genedb.org/Page/trypanosoma_surface_ phylome). This cell surface phylome provided a detailed analysis of species-specific gene families and of gene gain and loss in shared families, aiding in the identification of surface proteins that may mediate specific aspects of pathogenesis and disease progression. In order to further discuss our results, we evaluated which proteins from the* T. brucei* cell surface phylome [35] were common to the list of twenty-four* T. brucei* immunodiagnostic antigens that were found by Sullivan et al. [34]. African trypanosome genomes contain large VSG gene families [32, 33], but monoallelic expression of a single gene is ensured because transcription is restricted to telomeric VSG expression sites (ES) [36–38]. Several other expression site-associated Genes (ESAG1-12) [39–41] are located in the ES and are cotranscribed with the active VSG [32, 42]. The product for the* T. brucei* ESAG3 gene (Tb927.2.2020) has been reported as a protein of 44.2 kDa that contains two putative transmembrane helices. As ESAG3 is an integral membrane protein that was selectively recognized by* T. brucei gambiense* infection IgG, and most membrane-embedded proteins have posttranslational modifications (e.g., glycosylations) that will increase their apparent size by SDS-PAGE, we suggest that the* T. evansi* orthologous gene product for ESAG3 might correspond to the 53 kDa cross-reacting antigen that was recognized here by anti-*T. vivax* antibodies from infected bovines. In fact, two potential N-glycosylation sites were detected in the* T. brucei* ESAG3 protein using the NetNGlyc 1.0 server (http://www.cbs.dtu.dk/services/NetNGlyc/, R. Gupta, E. Jung, S. Brunak, 2004. Prediction of N-glycosylation sites in human proteins, manuscript in preparation). Yadav et al. [43] also identified a 52–55 kDa cluster of polypeptides from* T. evansi* as immunodominant antigens by Western blot using serum from an experimentally infected equine. Interestingly, two ESAG3 orthologous genes have been reported in the* T. vivax* genome (TvY486 0042500 and Tvy486 0043380). A similar analysis yielded the gene for the hypothetical protein 4180 (Tb927.6.4180), which corresponds to a transmembrane protein of 16,317 Da and was also identified as an immunodiagnostic antigen since it bound to the infection IgG fraction. A potential N-glycosylation site was also detected in the Tb927.6.4180 protein. Thus, we proposed that the* T. evansi* orthologous gene product for the hypothetical protein 4180 may correspond to the polypeptide band of 27 kDa that exhibited cross-reactivity with* T. vivax*. An orthologous gene for the hypothetical protein 4180 has also been reported in the* T. vivax* genome (TvY486 0603610).

By only using the* T. brucei* cell surface phylome generated by Jackson et al. [35], three additional genes that encode for hypothetical proteins of similar sizes, and contain transmembrane regions, were also found (Tb927.8.7720 of 24.9 kDa; Tb927.6.380 of 33.9 kDa; and Tb927.4.5070 of 51.3 kDa). Since the molecular masses of these hypothetical proteins coincide approximately with those of the* T. evansi* antigens that displayed cross-reactivity with* T. vivax *(27 kDa, 31 kDa, and 53 kDa), they might correspond to the* T. evansi* orthologous genes that encode for these hypothetical proteins. Yet, none of these genes were identified within the group of the* T. brucei* immunodiagnostic antigens reported by Sullivan et al. [34]. Genes Tb927.8.7720 and Tb927.4.5070 appeared to be specific for* T. brucei*; however, an ortholog for gene Tb927.6.380 has also been reported in the* T. vivax* genome (TvY486 0600040).* T. brucei* contains a number of other cell-surface immunodiagnostic antigens that were proteomically selected by Sullivan et al. [34], such as the gene related to ESAG (GRESAG) 4, which encodes for an adenylyl cyclase enzyme [44], and invariant surface glycoproteins (ISG) with molecular masses of 75 kDa, 65 kDa, and 64 kDa. However, the sizes of these proteins did not match with any of the integral membrane antigenic proteins from* T*.* evansi* that were identified here by their immunological cross-reactivity with* T. vivax*. Various members of the twenty-four* T. brucei* immunodiagnostic antigens reported by Sullivan et al. [34], which must also exist in* T. evansi*, possess comparable molecular masses to polypeptide bands identified in this work, for example, ESAG6 and ESAG7, which encoded for transferrin receptor subunits of 44,221 Da and 38,433 Da, respectively. However, ESAG6 and ESAG7 contain GPI anchor motifs that must be cleaved by the parasite GPI-PLC during the extraction steps, producing their corresponding soluble released forms. Consequently, ESAG6 and ESAG7 cannot be found in the SDS-solubilized particulate fraction of* T. evansi*.

Since previous results have demonstrated that one antigen may not be sufficient for diagnostic purposes, we strongly feel that a pool of antigens should be evaluated to develop a good immunodiagnosis assay. The identification and characterization of these antigens could serve not only for diagnosis, but also for prophylaxis and chemotherapy against bovine trypanosomosis. Moreover, the identification of the antigenic proteins that lead to the cross-reactivity between these two trypanosomes could help to better understand the evolution of these parasites. The specificity and sensitivity of all these cross-reacting antigens, either individually or in groups, are parameters being evaluated at the present time, in order to establish the possibility of utilizing them as tools for the serological immunodiagnosis of bovine trypanosomosis. However, differences in antigen expression among parasite isolates or immunogenetic variations among hosts could determine the ability for antigen recognition by the various animal species.

## 5. Conclusions

Based on the cross-reaction between* T. evansi* and* T. vivax*, the use of* T. evansi* antigens for the diagnosis of* T. vivax* infections represents an excellent alternative for laboratories lacking the facilities to produce* T. vivax* antigens. Three membrane-associated integral polypeptide bands from* T. evansi*, possessing apparent molecular masses of 27, 31, and 53 kDa, were partially purified here by centrifugation on linear sucrose gradients and were proven to be antigens that display cross-reactivity with* T. vivax*. These* T. evansi* polypeptides are attractive candidates to be considered as tools for immunodiagnosis of the trypanosomosis caused by* T. vivax*.

## Figures and Tables

**Table 1:** Identification of sera from experimentally infected animals.

Sample number	Sera name	Host	Infection agent	Isolate
1	B-103	Bovine	*T. vivax *	LIEM-176
2	B-173	Bovine	*T. vivax *	LIEM-176
3	B-303	Bovine	*T. vivax *	LIEM-176
4	H-TEVA1	Horse	*T. evansi *	TEVA1
5	H-TeApEF	Horse	*T. evansi *	TeAp-ElFrio01

**Table 2:** Identification of sera from field animals.

Sample number	Sera name	Natural host	Infection state	Infection agent	Locality
1	B-LC12	Bovine	Infected	*T. vivax *	La Candelaria Farm, Guárico State, Venezuela
2	B-LC29	Bovine	Not infected	—	La Candelaria Farm, Guárico State, Venezuela
3	B-LC31	Bovine	Infected	*T. vivax *	La Candelaria Farm, Guárico State, Venezuela
4	B-LC43	Bovine	Infected	*T. vivax *	La Candelaria Farm, Guárico State, Venezuela
5	B-LC54	Bovine	Infected	*T. vivax *	La Candelaria Farm, Guárico State, Venezuela
6	B-LC69	Bovine	Not infected	—	La Candelaria Farm, Guárico State, Venezuela
7	B-LL19	Bovine	Infected	*T. vivax *	La Loma Farm, Guárico State, Venezuela
8	B-LE14	Bovine	Infected	*T. vivax *	La Esperanza Farm, Guárico State, Venezuela
9	B-P13	Bovine	Infected	*T. vivax *	Paradero Farm, Guárico State, Venezuela
10	B-F5315	Bovine	Not infected	—	France
11	B-F5683	Bovine	Not infected	—	France
12	H-LR	Horse	Not infected	—	La Rinconada Racetrack, Caracas, Venezuela
13	H-N/D	Horse	Infected	*T. evansi *	N/D*, Apure State, Venezuela

*N/D: not determined.
